# Protocol for a hybrid type 3 effectiveness-implementation trial of a pragmatic individual-level implementation strategy for supporting school-based prevention programming

**DOI:** 10.1186/s13012-023-01330-y

**Published:** 2024-01-02

**Authors:** Aaron R. Lyon, Clayton R. Cook, Madeline Larson, Maria L. Hugh, Alex Dopp, Corinne Hamlin, Peter Reinke, Mahasweta Bose, Amy Law, Roger Goosey, Annie Goerdt, Nicole Morrell, Alisha Wackerle-Hollman, Michael D. Pullmann

**Affiliations:** 1 University of Washington, 6200 NE 74Th Street, Suite 100, Seattle, WA 98115 USA; 2 Character Strong, 4227 S Meridian, Puyallup, WA 98373 USA; 3 University of Kansas, 1122 W Campus Rd, Lawrence, KS 66045 USA; 4 RAND Corporation, 1776 Main Street, Santa Monica, CA 91604 USA; 5 University of Minnesota, 1954 Buford Avenue, Suite 425, St. Paul, MN 55108 USA

**Keywords:** Individual determinants, Implementation strategy, Theory of planned behavior, Health action process approach, Education sector, Mental and behavioral health

## Abstract

**Background:**

For approximately one in five children who have social, emotional, and behavioral (SEB) challenges, accessible evidence-based prevention practices (EBPPs) are critical. In the USA, schools are the primary setting for children’s SEB service delivery. Still, EBPPs are rarely adopted and implemented by front-line educators (e.g., teachers) with sufficient fidelity to see effects. Given that individual behavior change is ultimately required for successful implementation, focusing on individual-level processes holds promise as a parsimonious approach to enhance impact. Beliefs and Attitudes for Successful Implementation in Schools for Teachers (BASIS-T) is a pragmatic, multifaceted pre-implementation strategy targeting volitional and motivational mechanisms of educators’ behavior change to enhance implementation and student SEB outcomes. This study protocol describes a hybrid type 3 effectiveness-implementation trial designed to evaluate the main effects, mediators, and moderators of the BASIS-T implementation strategy as applied to Positive Greetings at the Door, a universal school-based EBPP previously demonstrated to reduce student disruptive behavior and increase academic engagement.

**Methods:**

This project uses a blocked randomized cohort design with an active comparison control (ACC) condition. We will recruit and include approximately 276 teachers from 46 schools randomly assigned to BASIS-T or ACC conditions. Aim 1 will evaluate the main effects of BASIS-T on proximal implementation mechanisms (attitudes, subjective norms, self-efficacy, intentions to implement, and maintenance self-efficacy), implementation outcomes (adoption, reach, fidelity, and sustainment), and child outcomes (SEB, attendance, discipline, achievement). Aim 2 will examine how, for whom, under what conditions, and how efficiently BASIS-T works, specifically by testing whether the effects of BASIS-T on child outcomes are (a) mediated via its putative mechanisms of behavior change, (b) moderated by teacher factors or school contextual factors, and (c) cost-effective.

**Discussion:**

This study will provide a rigorous test of BASIS-T—a pragmatic, theory-driven, and generalizable implementation strategy designed to target theoretically-derived motivational mechanisms—to increase the yield of standard EBPP training and support strategies.

**Trial registration:**

ClinicalTrials.gov ID: NCT05989568. Registered on May 30, 2023.

**Supplementary Information:**

The online version contains supplementary material available at 10.1186/s13012-023-01330-y.

Contributions to the literature

- Evidence-based prevention programs are often not adopted or delivered with sufficient fidelity to address children’s social, emotional, and behavioral health needs, even when conducive organizational supports are in place.
- This study tests a parsimonious and pragmatic individually focused implementation strategy delivered to frontline professionals (teachers) to enhance their implementation of an evidence-based prevention program. We will evaluate the effects on proximal outcomes/mechanisms (e.g., attitudes) as well as implementation (e.g., adoption) and children’s outcomes (e.g., social, emotional, and behavioral status).
- Findings will fill a gap in the literature surrounding the utility of pragmatic, individual-level strategies for preventive interventions as well as the variables (mechanisms) through which the strategies operate, and under which conditions the strategies work.

## Background

### Addressing children’s social, emotional and behavioral health

At least one in five children experience social, emotional, and behavioral/mental health (SEB) challenges [1], making accessible SEB prevention and intervention programming a high priority. When children experience SEB challenges, they are at an increased risk of academic and social difficulties in school and long-term experience with the judicial system, substance use problems, and unemployment [1]. In contrast, children who receive preventive SEB support experience social and academic gains into adulthood [2–6]). Given the widespread need to address children’s SEB, exacerbated by the COVID-19 pandemic [7, 8], accessible prevention programming is a high priority.

### School-based prevention

Schools are the most common setting in which children and adolescents in the USA receive both preventive and indicated care for SEB concerns [9–11]. As a result, school-delivered SEB practices have increasingly been prioritized in policy and legislation [12–14]. In particular, there are a variety of universal evidence-based prevention practices (EBPPs) that exist to address children’s SEB challenges [15, 16]. Among these are high-quality, effective, universal EBPPs delivered at the classroom or school level to support student SEB health [17–20].

### Need for improved implementation supports

As with other service sectors, EBPPs for school settings are adopted inconsistently and frequently delivered with poor fidelity [21–23]. In the education sector, this implementation gap has been resistant to change despite intervention at federal and state policy levels [24]. Even studies of quality implementation strategies, such as coaching and consultation, demonstrate that many EBPPs fail to be adopted by school-based implementers [25–27]. As a result, the potential public health impact of SEB-focused EBPPs is greatly diminished [28].

### Implementation determinants in schools

Like other health service sectors, schools are multi-level implementation contexts with myriad priorities, decision-makers, implementers, and recipients of the intervention. Across sectors, organizational influences on implementation have been the subject of considerable research, but creating organizational change is time-consuming and expensive, often lasting years [29]. Furthermore, even with appropriate implementation support to address organizational-level barriers or enact organizational facilitators, educators’ EBPP implementation can still be stilted.

Most implementation frameworks include critical individual implementer factors [30, 31]. Indeed, front-line professionals—such as teachers—are ultimately responsible for the adoption and delivery of EBPPs and present their own set of implementation determinants such as attitudes, beliefs, and intentions to implement an EBPP [30, 32, 33]. Some school-based research has documented that individual-level determinants can be more predictive of EBPP implementation than organizational factors, such as climate or culture [32, 34], even within supportive implementation contexts [35, 36].

### Individual-level implementation strategies

Despite significant research on cornerstone implementation strategies such as training and consultation [37, 38], additional approaches are often needed to change behavior. Individual theories of behavior change can be leveraged to further facilitate EBPP use. The current study applies the theory of planned behavior (TPB) [39] and the health action process approach (HAPA) [40]. The TPB states that an individual’s *subjective norms* (their perceptions of the social importance of performing the behavior), *attitudes* (appraisals of the behavior), and *task self-efficacy* (perceived behavioral control and confidence in their ability to implement the behavior), cumulatively predict *intentions* to perform a behavior [41]. Intention is a strong predictor of behavior change [42–44]. The HAPA augments the TPB by (1) positing that the intention to engage in a behavior is influenced by one’s *outcome expectancies* and *perceived risks* (beliefs about the possible consequences of a behavior and risks of not engaging in a behavior, factors that we cluster with “attitudes” in the TPB) and (2) emphasizing individual *volition* (initial action planning and planning for coping with barriers) that increase an implementers’ maintenance self-efficacy (the belief that one is capable of overcoming barriers while implementing the behavior) and facilitate the link between intentions and behavior. The most common implementation strategies, such as workshops, coaching, and consultation, primarily target knowledge and skills while often neglecting to explicitly attend to norms, attitudes, intentions, and volition.

### Beliefs and attitudes for successful implementation in schools for teachers (BASIS-T)

**Fig. 1:**
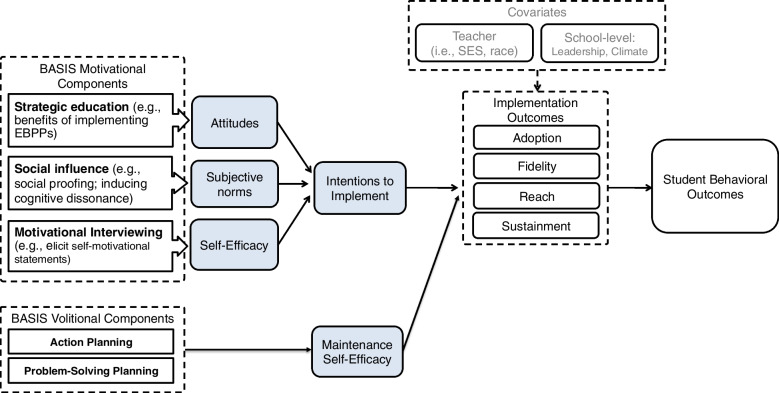
BASIS-T theory of change: components, hypothesized mechanisms of change and target outcomes

BASIS-T is designed to address individual-level mechanisms of behavior change (e.g., self-efficacy) often missing from standard EBPP training that relate to motivation prior to receiving training and volition after training. It is an EBPP-agnostic implementation strategy designed to be delivered within the preparation/adoption phase, immediately prior to active implementation [45]. BASIS-T targets behavioral intentions via improvement in attitudes, subjective norms, and self-efficacy. Our theory of change (Fig. 1) shows the core BASIS-T components, mechanisms of change (volitional and motivational), implementation outcomes, and resulting child SEB outcomes within the current study. The BASIS-T strategy is grounded in the TPB and in the HAPA strategies of *action planning* (specifying the “when,” “where”, and “how” of implementing the EBPP) and *problem-solving planning* (generating solutions to specific barriers that one anticipates encountering when adopting a new practice) to overcome barriers to implementation.

#### Preliminary BASIS-T studies

The BASIS strategy was developed via an iterative user-centered design approach [46]. Initial pilots of BASIS-T and a version designed for school-based mental health clinicians (BASIS-C) have demonstrated promise in enhancing participants’ attitudes, subjective norms, self-efficacy, and adoption [30, 33, 47]. The BASIS-T pilot study on which the current project is based was an attention-control randomized trial conducted with 82 elementary school teachers implementing a universal, classroom-based EBPP [48–50]. That study found a statistically significant positive impact on implementation task self-efficacy and outcome expectancy immediately after training and significantly less decline in task self-efficacy than attention control throughout the academic year [33]. Positive attitudes towards evidence-based practices decreased for both groups over time but with a marginal time trend in favor of less decrease for the BASIS-T condition (*p* = 0.08). Except for ownership/role, all other mechanisms (outcome expectancies, subjective norms, self-efficacy, and intentions to implement) deteriorated for both groups after the post-BASIS timepoint (i.e., during active implementation), yet all effect sizes were in favor of less deterioration in the BASIS-T condition. Significantly higher proportions of teachers in BASIS-T immediately adopted the EBPP (74% of BASIS-T condition, 40% of attention control). With marginal significance, fidelity to the EBPP remained steady for the BASIS-T group over time but deteriorated for attention control (*p* = 0.052), and the BASIS-T condition engaged in the EBPP more frequently (*p* = 0.097). A preliminary analysis estimated the cost of BASIS-T at $256 per teacher based on time and material costs. Notwithstanding the promise of these findings, this was an underpowered pilot trial designed to lay a foundation for the current study.

### Objectives and aims

This project will conduct a hybrid type 3 effectiveness-implementation randomized trial to evaluate the effects of BASIS-T on implementation mechanisms and outcomes when applied to positive greetings at the door (PGD), a low-burden, universal EBPP that has been found to reduce disruptive behavior and increase academic engagement [51–53], both important indicators of positive SEB functioning [54–57]. We will also examine for whom, under what conditions, and how efficiently BASIS-T works to improve outcomes.

PGD is a preventive classroom management strategy based on three major themes: (a) classroom climate, (b) pre-correction, and (c) positive reinforcement [53]. PGD has been found to be well aligned with school settings and effective at addressing SEB needs. Multiple studies have found increases in on-task behavior in middle school students, and reductions in latency-to-task engagement in high school students [51, 52]. These findings were replicated in a longitudinal randomized controlled efficacy trial conducted with 203 students across 10 classrooms, finding improvements in academic engagement and decreases in disruptive behavior [53]. However, consistent with more general research on universal EBPPs, results also suggested that some of the teachers delivering PGD struggled with initial adoption, with two of the five teachers in the PGD condition (40%) requiring extra consultative support due to initial low levels of implementation.

Aim 1: Experimentally evaluate the effects of BASIS-T versus active comparison control (ACC)

Aim 1 will evaluate the main effects of BASIS-T on proximal mechanisms (attitudes, subjective norms, self-efficacy, intentions to implement, and maintenance self-efficacy), implementation outcomes (adoption, reach, fidelity, and sustainment), and student outcomes (classroom aggregated grades, test scores, attendance, and teacher ratings of classroom on-task behavior, disruptive behavior, and prosocial behavior).

*Research question (RQ) 1a.* Is BASIS-T more effective than the ACC condition at producing changes in proximal mechanisms of behavior change?

*RQ 1b.* Is BASIS-T more effective than the ACC condition in promoting implementation outcomes?

*RQ 1c.* Is BASIS-T more effective than the ACC condition in promoting meaningful changes in student SEB and academic outcomes?

Aim 2: Evaluate how, for whom, under what conditions, and how efficiently BASIS-T works to improve outcomes

Aim 2 will evaluate the effects of BASIS-T on student outcomes via the mechanisms of implementation behavior change and if those effects are moderated by teacher factors and school contextual factors. We will also explore how mechanisms are linked to implementation outcomes for “hypothesis-defying residuals” (i.e., teachers whose attitudes, subjective norms, and self-efficacy surrounding EBPP implementation are inconsistent with their documented implementation behaviors).

*RQ 2a*. Are the effects of BASIS-T mediated via mechanisms of behavior change?

*RQ 2b*. Are the effects of BASIS-T on implementation and student outcomes moderated by teacher-level factors (e.g., demographics, stress, baseline intentions to implement) and school-level factors (e.g., geographic location, school demographics, supportive leadership, implementation climate)?

*RQ 2c*. What explains “residual” teachers whose implementation behaviors are not accounted for by the mediation model?

*RQ 2d.* What are the costs and cost-effectiveness of BASIS-T?

## Method

**Fig. 2:**
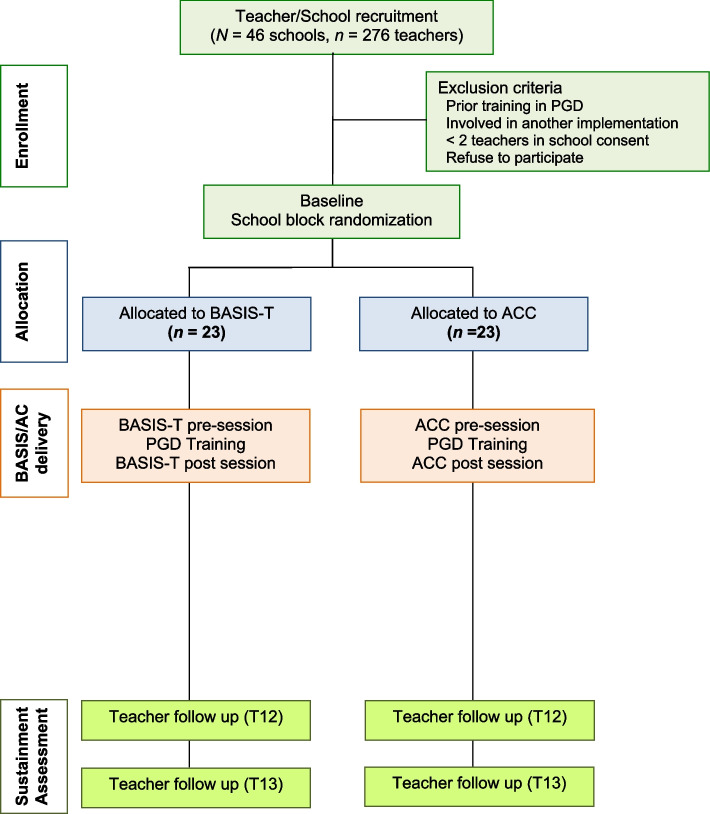
CONSORT diagram

This hybrid type 3 effectiveness-implementation trial employs a blocked randomized cohort design with an active comparison control (ACC) condition to provide a rigorous initial test of the efficacy of BASIS-T in authentic elementary school settings (see Additional file 1 for SPIRIT checklist). Schools will be randomized to BASIS-T or ACC conditions (see Fig. 2) and data will be gathered at the teacher/classroom level. There will be two cohorts of participants—one for each of two academic years—and multiple time points of data collection over 18 months across implementation and sustainment phases. Institutional review board approval has been obtained (Additional file 2), which includes plans for de-identification and secure data storage as well as tracking and reporting of adverse events or protocol modifications if needed.

### Participants and recruitment

Teachers from schools in the USA will be recruited to participate. Inclusion criteria include being a teacher at an elementary (typically K–5th grade or K–8th) school and not having been trained or supervised in delivering PGD in the past 5 years. We will recruit approximately 276 teachers from 46 elementary schools in the USA. The final balance of teachers and schools is dependent on recruitment, with the goal of meeting minimum statistical power. Schools will be approached to participate via multiple routes, including leveraging existing relationships and networks, educational listservs, and posting on social media. Interested school representatives may respond using the interest survey linked in flyers or by contacting the research team via email. The generalizer (generalizer.org), a free, web-based tool for selecting schools for randomized controlled designs that are statistically representative of a chosen inference population, will also be used to help assure the representativeness of our sample [58]. Strata will be created on urbanicity, school race/ethnicity, school percent female, school percent free and reduced lunch, school size, school percentage English language learners, and the number of schools in the district.

Once schools have been selected, teacher recruitment will proceed for both cohorts of schools with the assistance of site administrators. Principals will provide us with the email addresses of teachers in their schools. Research staff or school leadership will contact eligible participants by email or phone to describe the purpose of the study, research procedures, and incentives. Informed consent will be collected online prior to training (Additional file 3) and teachers will be free to decline participation. Monetary incentives will be provided to participating teachers and schools.

### Randomization

This study will employ a randomized cluster-blocked cohort design with random assignment at the school level to eliminate the possibility of condition contamination among teachers. Schools will be blocked within the district by a number of teachers participating in the study, school enrollment, % of non-White students, % of students who qualify for free/reduced lunch, and mean teacher baseline BASIS-T mechanisms of change (e.g., self-efficacy). These variables were chosen because they are associated with EBPP use and student academic outcomes [59]. We will create matched pairs using the nearest neighbor approach [60] and randomly assign schools within pairs to condition. Randomization will occur to the greatest extent possible, although there may be some situations where trainers in only one condition are available when school staff are available to be trained; these situations will be considered essentially random. School personnel and participants will be masked to condition.

### Intervention

All participants will receive standard tele-delivered training on PGD from educational consultants with expertise in its implementation and in school-based EBPP training more generally. PGD is a proactive classroom management strategy [61] that takes a prevention-based approach to responding to behavioral needs in the classroom [53]. Research shows that PGD can increase student-level outcomes such as on-task behavior and decrease disruptive behavior [51–53]. PGD was designed to facilitate smoother transitions in the classroom by (a) connecting with each student by greeting them by name, (b) using pre-corrective statements with the entire class to communicate expected behaviors for transitions into the classroom, (c) providing specific pre-corrective statements privately to individual students who have difficulty self-regulating their behavior, and (d) providing specific praise and encouragement to students to reinforce desired behaviors [53].

### Implementation conditions

#### BASIS-T strategy

##### BASIS-T motivational components

The BASIS-T implementation strategy integrates three core motivational components (Table 1). First, the BASIS-T facilitator provides strategic education about implementing EBPP and overcoming barriers via maintaining an internal locus of control to improve attitudes. The second component is social influence techniques to alter perceptions of subjective norms, which consists of two broad approaches: (1) social proofing messages using data or testimonials to describe the behavior or attitudes of others and (2) techniques to induce cognitive dissonance. Social proofs have been used to reduce problem behaviors, including alcohol/drug use and disordered eating behaviors [62–64]. Techniques to induce cognitive dissonance operate on the premise that individuals strive for consistency between attitudes and actions [65]. Desired behaviors can be increased by evoking commitments that are active, public (vs. private), and voluntary (vs. coerced) [66]. Third, motivational interviewing (MI) is used to enhance self-efficacy. MI is a collaborative, person-centered approach to elicit and strengthen motivation to change [67]. MI has been adapted as a brief intervention with strong evidence, feasibility, and acceptability among school-based mental health clinicians [68]; shown to improve self-efficacy and implementation among teachers and primary care providers [68, 69]; and used in group contexts to promote change [47]. The BASIS-T facilitator uses group MI techniques by adopting an empathic, non-directive, and person-centered style to elicit self-motivational statements, encourage discussion of potential changes (“change talk”) and enhance self-efficacy.

**Table 1:** BASIS-T strategy components

**Motivational components** **(TBP mechanisms)**	**Strategic education (attitudes)**
	Connecting EBPP to student success
	Maintaining an internal locus of control
	**Social influence (subjective norms)**
	Providing normative information
	Testimonials from experts
	Testimonials from similar others
	Evoking public commitments
	**Motivational interviewing (self-efficacy)**
	Elicit-provide-elicit structure
	Professional values clarification activity
	Pros and cons activity to illicit change talk
	Anticipating implementation barriers
	Values-directed goal setting
	“Ruler questions” (e.g., how confident are you)
**Volitional components** **(HAPA mechanism)**	**Action planning and problem-solving planning** **(Maintenance self-efficacy)**
	Action planning to initiate implementation
	Problem-solving planning to overcome barriers

##### BASIS-T volitional components

To address the intention-behavior gap, BASIS-T includes volitional planning interventions to increase the likelihood that teachers will maintain their self-efficacy by enacting specific implementation behaviors associated with an EBPP. These strategies have been shown to help people enact health behaviors they are already motivated to perform [70–75]. Moreover, these planning interventions have demonstrated success in improving teacher adoption of interventions for student behavior [76]. These interventions support the translation of intentions into actions through detailed planning of how to perform desired behaviors in specific contexts. Solutions are generated to situational and internal (e.g., cognitive) barriers to facilitate follow-through with the action plan. In combination, action planning and problem-solving planning increase the likelihood of behavior change [77].

##### BASIS-T structure

BASIS-T is delivered in a group-based format shortly before and after receiving EBPP (e.g., PGD) training. BASIS-T will be delivered via tele-facilitation (via Zoom or another similar video conferencing platform), pre-recorded video content, and electronic sharing of documents to promote scalability. BASIS-T facilitators are experienced school-based professionals. A *pre-training session* (75 min) targets attitudes, social norms, and perceived behavioral control. The pre-training opens with the facilitator engaging teachers in an activity to clarify their professional values (an MI component). The facilitator uses open-ended questions to elicit change talk and reflects, summarizes, and draws themes across participant responses. The pre-training session is designed to help participants (a) explore their professional values and goals and make connections between those and EBPP training opportunities, (b) link EBPP delivery to improved outcomes for students, and (c) recognize common cognitive shortcuts that leave individuals vulnerable to adopting non-EBPPs. Teachers collaboratively develop an individualized menu of potential solutions to implementation barriers from which they can select when encountering challenges and set value-congruent goals related to the upcoming EBPP training.

The BASIS-T *post-training session* (75 min) provides protected time and a structured experience to develop action plans and problem-solving plans. Teachers will be provided with an action planning template to detail what PGD components they will use, how, with whom, where/when, and the environmental cues and resources needed to serve as prompts to deliver PGD with fidelity. The problem-solving plan involves teachers anticipating situational barriers and generating solutions to overcome those barriers to develop personalized “if–then” plans for dealing with specific barriers. Teachers share their plans with colleagues to receive input and feedback and to publicly set values-based goals for implementation.

##### BASIS-T fidelity

The BASIS-T pre- and post-training intervention sessions will be recorded and independently coded by two trained research assistants with disagreements resolved through consensus dialogue [78, 79]. The research assistants will use the BASIS-T fidelity tool [47] to assess the fidelity of its delivery.

#### Active comparison control

Teachers assigned to the ACC will receive pre- and post-training experiences designed to mirror those received in the BASIS-T condition. These training experiences will be virtual (again via Zoom or similar) and approximately the same length as BASIS-T but will not contain any of the BASIS-T content or mechanisms of change. The ACC pre-training experience will define, describe, and advocate for EBPP implementation and fidelity of EBPP use in schools. Content will be delivered in modes that mirror that of BASIS-T with video content, workbooks, and didactic training. The ACC thus controls for dose, information provided, and delivery mode effects. Some trainers will provide ACC and BASIS-T to reduce the potential for trainer effects.

#### Teacher and school data collection

Teacher data collection will span both active implementation and sustainment phases (18 months per cohort). Data will include teacher quantitative web-based surveys and qualitative interviews, each of which will be incentivized. To promote data integrity, key items will be forced to choose to prevent unplanned missingness and out-of-range responses. All data will be de-identified and stored securely. Detailed information about all study measures (including citations) can be found in Additional file 4.

#### Quantitative surveys

Participants will complete secure web-based surveys at 13 timepoints spanning three phases: preparation (times 1–4), active implementation (times 5–11), and sustainment (times 12–13 follow-up during the next academic year) [45]. Time 1 will occur during informed consent, time 2 in the days immediately before BASIS-T/ACC pre-training, time 3 immediately after pre-training, time 4 immediately after post-training, time 5 two weeks after training, time 6 four weeks after training, times 7–11 monthly until the end of the academic year, time 12 at the beginning of the subsequent academic year, and time 13 during the spring of that academic year. Teachers will self-report demographics, perceptions about BASIS-T (when relevant) and PGD training, BASIS-T mechanisms, implementation outcomes, organizational moderators (implementation climate, leadership), and time and resources used for PGD implementation. From times 7–11, to reduce the respondent burden we will use a random item planned missingness design for measures of attitudes (1 item selected per subscale), action self-efficacy (2 items selected), and subjective norms (1 item selected per each scale).

Data collection regarding students will focus on classroom behavior, behavioral discipline, attendance, and academic performance and will be collected in aggregate at the classroom level, without individual identifiers. Teachers will complete the first secure online survey on student classroom behavior at time 4, as earlier data collection would prohibit teachers from having an adequate sample of student behavior. At the end of each school year, academic and behavioral data (attendance, disciplinary actions, grades, and standardized test scores) will be requested for all students who were in classrooms of participating teachers; these data will be obtained in aggregate at the classroom level and no individual identifiers will be provided to the research team.

#### PGD fidelity

Teachers will complete self-reported PGD fidelity assessments, aligned with recommendations to gather reliable and valid data, monthly in the time 4–13 surveys. Additionally, observations to assess the PGD fidelity of the implementing teachers will be conducted by trained school-based personnel using a standard PGD fidelity tool.

#### Qualitative interviews

Teachers whose implementation behavior is insufficiently accounted for by our mediation model (e.g., teachers with favorable implementation outcomes, but who demonstrate low levels of TPB constructs and/or teachers with low implementation outcomes, but high TPB constructs) will be invited to a qualitative interview at the end of the active implementation phase to explore additional implementation determinants. These teachers will be identified at the end of their first year of participation based on the results of statistical modeling, balanced between adopters and non-adopters, and BASIS-T and ACC conditions (15 to 19 interviewees total). Semi-structured phone interviews (approximately 60 min) will be conducted at a convenient time for identified teachers and audio-recorded for transcription and coding purposes.

#### Cost assessments

Cost data collection will occur with all participating teachers to capture major costs of PGD delivery with and without BASIS-T, using activity-based costing to focus on key expenses (e.g., teacher and staff time, materials) [80, 81]. We will measure costs from the payor (i.e., school system) perspective, since the primary costs and associated decision-making would be within the implementing school system in real-world implementation. These data will be collected along with other study measures depending on how often an item needs to be measured (e.g., one-time training prep in time 4 vs. PGD delivery collected monthly alongside the fidelity measures). Following expert guidance to use mixed methods in implementation cost studies [82], we will include open-ended items in each survey asking teachers to identify unexpected resources they have needed for BASIS-T or PGD. ACC costs will be excluded from the cost estimate for the comparison condition; PGD implementation-as-usual is the ideal counterfactual for the cost-effectiveness of BASIS-T because it represents “business as usual” for PGD and, unlike BASIS-T, ACC would never be delivered outside of a research project. To develop complete cost estimates for BASIS-T versus PGD implementation-as-usual, we will combine the teacher-reported data with information from other sources, such as BASIS-T training records and meetings with school partners.

#### Data analytic plan

Basic data screening and descriptives will be conducted for all quantitative variables. We will explore and statistically adjust for baseline equivalence between conditions on all individual outcomes and all school, teacher, and student variables following established guidelines [83]. For all longitudinal modeling, the statistical adjustment will use baseline intercepts as random terms; for dissimilar outcome domains, non-equivalent baseline variables will be included as covariates. Data missing at random will be modeled using full information maximum likelihood estimation or multiple imputation as appropriate.

Unless otherwise indicated, quantitative analyses will employ mixed-effects models using time points nested within classrooms within the school. Standard model-building procedures will be used [84, 85]. Classroom and time trends will be allowed to randomly vary. Piecewise time models will estimate slopes from T1 to T4 (training), T5 through T6 (first month of post-training implementation), T7–T11 (first academic year), and T12–T13 (sustainment during the subsequent year). Variables for condition and condition x time will be added, and iterative models with possible control variables will be tested. Covariates not contributing at *p* < 0.10 based on likelihood ratio tests will be removed. Level 2 and 3 predictors will be fit and excluded for the same reasons. We will obtain estimates of whether there were statistically significant differences among the groups on the rate of change over time (i.e., slope) and whether there are statistically significant group differences in the average score on each outcome variable at the final timepoint of each piecewise segment. Models will be generalized, with appropriate link functions (e.g., log-link, Poisson) applied based on distributional form (e.g., dichotomous, zero-inflation). Estimation will be fit using full maximum likelihood. Models will be assessed for possible violations of assumptions. Goodness-of-fit will be evaluated using likelihood ratios, deviance statistics, and fit criteria. The inference will be evaluated relative to *p* < 0.05. For RQs with multiple DVs, we will adjust for the false discovery rate using Benjamini and Hochberg’s procedure [86].

We will use thegeneralizer.org to compare our sample to national demographics to examine the generalizability of our findings [58]. If not generalizable, we will use inverse probability weighting to increase the representativeness of estimates [87].

*RQ 1a*: A series of 3-level piecewise mixed effects models will be used to test our primary hypotheses: (1) the BASIS-T condition will show steeper gains than the ACC condition from T1 to T2 (e.g., more favorable attitudes towards EBPPs, increased social norms; enhanced task and maintenance self-efficacy; stronger intentions to implement) and (2) both groups will decline after training (T3–T5) as has been found in past TPB research [88], and in the BASIS-T pilot trial [33], but the BASIS-T condition will have longer sustained between-condition effects after training from T3 to T13. Dependent variables will include subscale scores on attitudes, social norms, self-efficacy, intentions, and maintenance self-efficacy.

*RQ 1b*: BASIS-T impact on behavioral implementation outcomes will be tested in two ways. First, a mixed effects model will examine whether the proportion of teachers in the BASIS-T condition who adopt PGD (i.e., initiate PGD) is higher than the proportion in the ACC condition. Second, Kaplan–Meier time-to-event analyses will be used to compare conditions on the number of days between training and PGD initiation. Reach will be analyzed using mixed effects models comparing BASIS-T vs. ACC on the proportion of students in each classroom (out of those eligible based on whether their classroom teacher participated in PGD training) who received PGD practices. Impact on PGD fidelity will be analyzed using mixed effects models with sessions within teachers, testing for the main effects of condition on adherence and participant responsiveness ratings derived from both PGD observational and self-report fidelity data. We will test the effects of BASIS-T on PGD sustainment and delayed implementation using the multilevel longitudinal analytic approach described for RQ1a.

*RQ1c*: BASIS-T impact on student SEB and academic outcomes will be tested via mixed effects multilevel models as described in RQ1a, with academic data aggregated to the classroom level. Dependent variables will include post-intervention scores on teacher ratings of student behavior.

*RQ2a*: Whether mechanisms of behavior change mediate the impact of BASIS-T on implementation outcomes will be analyzed using path analysis [89] extending traditional mediation modeling to a multi-level framework for nested data [90, 91].

*RQ2b*: To test whether the effect of BASIS-T on implementation and student outcomes is moderated by teacher factors (e.g., demographics, baseline intentions to implement) and school-level factors, we will add moderators and interaction terms to the analytic approach described in RQs 1a-c.

*RQ2c*: To explore what explains “residual” teachers whose implementation behaviors are not accounted for by the mediation model, we will analyze qualitative interviews with teachers who have a difference between predicted (based on BASIS-T putative mechanisms) and actual implementation behavior of ≥ 1 SD. Data will be coded using an integrated directed and conventional content analysis [92] approach as certain codes will be conceptualized during the interview guide development and driven by the exploration, preparation, implementation, sustainment [93] framework (i.e., deductive approach) which will allow for the examination of influences on implementation across multiple levels and phases. Other codes will be developed through a close reading of an initial subset of transcripts (i.e., inductive approach). These themes will provide a way of identifying and understanding the most salient factors that impact implementation and extend beyond the existing BASIS-T mechanisms and theory of change. After a stable set of codes is developed, a consensus process will be used in which all reviewers independently code and compare their coding to arrive at consensus judgments through open dialogue [78, 79, 94].

*RQ2d*: We will process cost data by assigning monetary values to each cost. We will use CostOut [95], a web-based program for conducting cost-effectiveness analysis in education, to identify nationally representative unit prices for ingredients. For qualitative data, we will rapidly analyze responses on an ongoing basis [96, 97] and incorporate newly identified costs into future surveys for quantitative measurement.

Once cost data collection is complete, we will calculate the costs of BASIS-T versus PGD implementation-as-usual based on the unit price and amount of each cost category (e.g., hours spent, items purchased). We will use CostOut to standardize dollar values, including an index year for inflation; cost-of-living adjustments; and discounting costs from different years to account for preferencing of delayed over immediate costs [80, 95]. We will generate descriptive statistics describing typical costs (i.e., means, standard deviations) for each condition and incremental costs of BASIS-T over implementation as usual.

Once the cost analysis is complete, we will use CostOut to calculate the cost-effectiveness [81] of BASIS-T versus PGD implementation as usual. This will involve calculating a series of incremental cost-effectiveness ratios for each implementation and student outcome measure, representing the incremental costs of BASIS-T divided by its incremental benefit (i.e., effect size). Themes about BASIS-T mechanisms and outcomes from the qualitative teacher interviews will allow for mixed-method cost-effectiveness evaluation [82] in which participants’ views help determine whether the results were worth the cost. For both cost and cost-effectiveness analyses, we will conduct sensitivity analyses that vary key sources of uncertainty in the models to examine the robustness of our estimates [98, 99].

#### Power

Our planned sample, including attrition, will provide sufficient power to test linear effects on teacher- and classroom school-level variables with a minimum detectable effect size (MDES) of small to medium effects, *d* = 0.37. We used PowerUp! [100] assuming clustering, final samples of 46 schools evenly randomized to condition, 6 teachers per school (after 15% attrition), school ICCs of 0.10 (consistent with our pilot data), and 13 timepoints. With the same assumptions as above, plus an assumption that 50% of the schools will be in a given moderator subgroup, our MDES for detecting any single school-level moderator variable is 0.48 and teacher-level moderator variable is 0.45. For mediator analyses, PowerUp! identifies power using the Sobel test, joint test, and Monte Carlo simulations. Across all tests, we will have a power of greater than 0.80 to detect reasonable and likely effects, based on our pilot trial and standard interpretations of effect sizes. We will detect an MDES equivalent to Cohen’s *d* = 0.60 for the treatment-mediator pathways (e.g., BASIS-T to implementation intention) which is lower than our actual obtained effect size from the pilot trial (which ranged from *d* = 0.61 to 1.16 for our primary mediators), an MDES of Pearson’s *r* = 0.3 for the mediator-outcome path (e.g., implementation intention to student behavior, a small effect), and an MDES of *d* = 0.10 for the direct path from treatment to student outcome.

## Discussion

### Innovation

This hybrid type 3 trial will contribute to the literature on pragmatic implementation strategies, as well as nascent but expanding literature on implementation mechanisms [101–103]. Aside from BASIS-T’s counterpart strategy, BASIS-C [104], no studies of strategies have been explicitly designed to impact TPB and HAPA mechanisms while testing those mechanisms via mediation models. Recent systematic reviews [103] indicate that much more work is necessary surrounding implementation strategy mechanisms to allow for the development of streamlined, pragmatic approaches to improving implementation outcomes that can be generalized across EBPPs. The current study also contributes significantly to theory-building in implementation by exploring factors beyond TPB and HAPA that help to explain hypothesis-defying residuals’ relationships between mechanisms and behavior.

In addition, this project will examine the costs and cost-effectiveness of the BASIS-T strategy. Cost-effectiveness is an important driver of adoption decisions, especially at system and policy levels [105]. Examination of cost-effectiveness is particularly critical for implementation strategies designed with pragmatism in mind. Efficient delivery and impact are key components of pragmatism, as are clear links between prioritized implementation determinants (e.g., self-efficacy in BASIS-T) and strategy components [106].

### Limitations

This study uses a block randomization approach in which all participating teachers in each school are randomized to either BASIS-T or the ACC condition. Although our team considered randomizing at the individual teacher level to align with the individual focus of the BASIS-T strategy, we opted against this because it presented a significant risk of contamination because of the extent to which some of BASIS-T’s putative mechanisms are likely to be socially influenced (especially subjective social norms).

## Conclusion and impact

The current study will provide evidence of the efficacy and cost-effectiveness of applying BASIS-T in an educational setting alongside EBP training to improve implementation outcomes. Trial results will be disseminated via publications, presentations via traditional (e.g., press releases) and social media, and through networks of practitioners. Positive findings from this trial would support the generalizability of BASIS-T to additional universal, school-based EBPPs for social, emotional, and behavioral health. More generally, if effective, it will add to the growing evidence for pragmatic implementation strategies and the mechanisms through which they operate.

### Supplementary Information

**Additional file 1.** SPIRIT 2013 Checklist.

**Additional file 2.** IRB Determination.

**Additional file 3.** Consent Form for Teachers.

**Additional file 4.** Detailed Study Measures [107–115].

## Data Availability

Please contact the lead author for more information.

## Abbreviations

ACC: Active control condition
BASIS-T: Beliefs and attitudes for successful implementation in schools
EBPP: Evidence-based prevention programs
HAPA: Health action process approach
MI: Motivational interviewing
MDES: Minimal detectable effect size
PGD: Positive greetings at the door
SEB: Social, emotional, and behavioral health
TAU: Treatment-as-usual
TPB: Theory of planned behavior
