# Supplementary material for: Protocol for a hybrid type 3 effectiveness-implementation trial of a pragmatic individual-level implementation strategy for supporting school-based prevention programming
Source: Implement Sci. 2024 Jan 2;19:2. doi: 10.1186/s13012-023-01330-y (PMC10763475; doi:10.1186/s13012-023-01330-y)
Supplement: Supplementary file 2 — Additional file 2. IRB Determination. [file 13012_2023_1330_MOESM2_ESM.pdf]

# DETERMINATION OF EXEMPT STATUS

May 4, 2023

Dear Aaron Lyon:

On 5/4/2023, the University of Washington Human Subjects Division (HSD) reviewed the following application:

| | |
|---|---|
| Type of Review: | Initial Study |
| Title of Study: | BASIS-T: Supporting Teachers in Implementing Classroom Practices to Build Better Student-Teacher Relationships |
| Investigator: | Aaron Lyon |
| IRB ID: | STUDY00017371 |
| Funding: | Name: Institute of Education Sciences (IES), Grant Office ID: N/A, Funding Source ID: R305A210241<br>Funding Title(s):<br>Pass-through institution(s): |

## Exempt Status

**HSD determined that your proposed activity is human subjects research that qualifies for exempt status (Category 2)**. This determination may or may not be based on the Limited IRB Review process.

- This determination is valid for the duration of your research.
- This means that your research is exempt from the federal human subjects regulations, including the requirement for IRB approval and continuing review.
- **Depending on the nature of your study, you may need to obtain other approvals or permissions to conduct your research. For example, you might need to apply for access to data or specimens (e.g., to obtain UW student data). Or, you might need to obtain permission from facilities managers to approach possible subjects or conduct research procedures in the facilities (e.g., Seattle School District; the Harborview Emergency Department).**
- HSD does not make determinations on behalf of other institutions. If other institutions are involved in the research, they may need to make their own determination or they may decide to be guided by our determination.
- Your study has a Certificate of Confidentiality (CoC) because you have federal funding or because you have applied for and received one from a federal agency. See this INFORMATION SHEET for a description of the CoC protections and responsibilities.

If you consider changes to the activities in the future and know that the changes will require HSD review (or you are not certain), you may request a review or new determination by submitting a Modification to this application. For information about what changes require a Modification, refer to the **GUIDANCE Exempt Research**.

HSD does not review or approve consent plans and consent materials for exempt research. Researchers are still responsible for providing subjects with information about the research prior to their agreement to participate. Refer to the **GUIDANCE Exempt Research** for details about what information should be provided. You may wish to use the optional **TEMPLATE Consent Exempt Research** as a guide.

Thank you for your commitment to ethical and responsible research. We wish you great success!

Sincerely,

Lindsey Westlake
Senior Administrator
206-897-1748 | scaggl@uw.edu
