# Supplementary material for: Protocol for a hybrid type 3 effectiveness-implementation trial of a pragmatic individual-level implementation strategy for supporting school-based prevention programming
Source: Implement Sci. 2024 Jan 2;19:2. doi: 10.1186/s13012-023-01330-y (PMC10763475; doi:10.1186/s13012-023-01330-y)
Supplement: Supplementary file 3 — Additional file 3. Consent Form for Teachers. [file 13012_2023_1330_MOESM3_ESM.docx]

**UNIVERSITY OF WASHINGTON**

**CONSENT FORM**

**Supporting Teachers in Implementing Classroom Practices to Build Better Student-Teacher Relationships**

Researchers:

Aaron Lyon, Ph.D., Professor, Department of Psychiatry, (206) 221-8604

Michael Pullman, Pd.D., Associate Professor, Department of Psychiatry, (206) 685-0408

We are asking you to be in a research study. This form gives you information to help you decide whether or not to be in the study. Being in the study is voluntary. Please read this carefully. You may ask any questions about the study. Then you can decide whether or not you want to be in the study.

**PURPOSE OF THE STUDY**

The purpose of this study is to explore the adoption and delivery of evidence-based prevention practices (EBPP) in elementary classrooms. More specifically, we want to examine the impact of a teacher-focused practice and its impact on teachers’ use of evidence-based classroom practices and programs with fidelity (that is, as planned with sufficient consistency, accuracy, and competency). We will be examining teachers’ experiences before, during, and after receiving training in EBPPs, as well as the impact of implementation of EBPPs on student outcomes.

**STUDY PROCEDURES**

This study will work with over 200 teachers from across the United States. We anticipate your participation will last for 18 months across two academic calendar years. During this time, you will take part in the following:

- You will receive virtual training on an evidence-based practice called Positive Greetings at the Door (PGD).
- In addition to receiving group-based, interactive training and consultation in PGD, you will also receive supplemental support before and after PGD training, involving presentations, discussions, and activities to support your use of PGD.
- You will be asked to complete brief, secure web-based surveys at 13 time points and receive compensation for each. Most of these surveys will take less than 15 minutes.
- You will be asked to participate in three 15-minute classroom observations, conducted by a member of the research team or your school administrative team trained by the study team.
- You may be asked to participate in an interview via phone or video conference call with a member of the research team. This interview would last up to 1 hour if you are invited.

**BENEFITS OF THE STUDY**

The primary direct benefit to you of participation is receiving free training on PGD You may also appreciate the opportunity to contribute to the larger body of knowledge related to improving services for students in schools. The research may lead to discoveries that enhance the understanding of how to successfully implement EBPPs in schools.

**RISKS, STRESS, OR DISCOMFORT**

A standard risk is a breach of confidentiality. Your classroom will be observed, which may cause temporary discomfort.

**CONFIDENTIALITY OF RESEARCH INFORMATION**

All of the information you provide will be confidential. We take steps to guard your privacy. Your name will not be used in the research: you will be assigned a study code. We will keep paper copies of study information in locked files that only the researchers can access. Some aspects of data collection (e.g., qualitative interviews) will be recorded and may be professionally transcribed by a company that provides secure data uploading and has adequate data protection measures in place. Digital data will be protected with encryption on secure servers accessible only to the research team. The transfer of audio/video files will be done via secure, HIPAA approved cloud services. All audio files will only be listened to by members of our study staff.

The researchers will write papers and make presentations that will educate the public about what they learn from the study. No names or other identifying information will be used in any papers or presentations that may result from this study. When our research team looks at or reports study findings, all answers will be combined with the answers of the other participants and no names will be used. Government or university staff members sometimes review studies such as this one to make sure they are being done safely and legally. If a review of this study takes place, your data may be examined. The reviewers will protect your privacy. If you have any questions, Dr. Lyon or the research staff will answer them at any time.

**USE OF INFORMATION AND SPECIMENS**

**Using Your Data in Future Research**

Consistent with IES policies, the information that we obtain from you for this study might be used for future studies with other authorized research teams. We will remove anything that might identify you from the data. If we do so, that information may then be used for future research studies or given to another investigator without getting additional permission from you.

**OTHER INFORMATION**

You may refuse to participate and you are free to withdraw from this study at any time without penalty or loss of benefits to which you are otherwise entitled. If you wish to withdraw, please contact the researcher listed on page 1 of this consent form.

Incentives for participation in study procedures will vary at each stage, all paid through an e-gift card. Incentives for completing the training may total to e-gift cards of $250 in value, while completing all surveys during study will earn $600 in e-gift cards. If you are selected to take part in a voluntary interview with research staff, you will earn a $100 e-gift card as an incentive.

Individual schools that participate in the study may earn up to $1,400, delivered via check, for building-level incentives.

If you earn $600 or more in payments from the University of Washington during this calendar year, the UW Financial Management Office will report this to the Internal Revenue Service as Miscellaneous Income.

A copy of the consent form will be emailed to you at an email address that you provide. It will be a “PDF” document. If you would prefer to receive a paper copy of the consent form at no cost to you, please contact the researcher listed on page 1 of this consent form.

Statement of Consent

This study has been explained to me. I volunteer to take part in this research. I have had a chance to ask questions. If I have questions later about the research, or if I have been harmed by participating in this study, I can contact one of the researchers listed on the first page of this consent form. If I have questions about my rights as a research subject, I can call the UW Human Subjects Division at (206) 543-0098. I will receive a copy of this consent form.

Printed name of subject Signature of subject Date
