# Supplementary material for: Protocol for a hybrid type 3 effectiveness-implementation trial of a pragmatic individual-level implementation strategy for supporting school-based prevention programming
Source: Implement Sci. 2024 Jan 2;19:2. doi: 10.1186/s13012-023-01330-y (PMC10763475; doi:10.1186/s13012-023-01330-y)
Supplement: Supplementary file 4 — Additional file 4. Detailed Study Measures [107–115]. [file 13012_2023_1330_MOESM4_ESM.docx]

**Additional File 4 – BASIS-T Study Measures**

| **Construct** | **Measure** | **Type** | **Informant** | **Time** |
| --- | --- | --- | --- | --- |
| *Demographics and Context* | | | | |
| Teacher demo-graphics | Race, sex, gender, years teaching, grades taught, education level, EBP experience, household income. | S | T | T1 |
| School character-istics | School size, racial composition, % eligible for free/reduced priced lunch, etc. | R | R | T1 |
| *Mediators (Mechanisms) and Moderators for BASIS-T and PGD* | | | | |
| Attitudes towards EBP | School Evidence-Based Practice Attitudes Scale, Teacher version (S-EBPAS): The school-adapted version of the Evidence-Based Attitudes Scale (EBPAS) [107] is a widely used 15-item tool designed to assess EBP attitudes with 4 subscales. Items are rated on a five-point scale (0 = “Not at All” to 4 = “To a Very Great Extent”). Internal reliabilities are adequate for the total score (alpha = .81) and the subscale scores: (1) appeal of EBPs (alpha = .77), (2) openness to new practices (alpha = .84), (3) likelihood of adopting an EBP (alpha = .60), and (4) perceived divergence between current practices and EBPs (alpha = .67). Construct validity has been demonstrated via factor structure replication in cross-cultural samples. Example items include “I would adopt an EBP if my youth would benefit from it”, “I would try a new EBP even if it were very different than what I am used to doing”. Respondents indicate the extent to which they agree with each statement on a scale of 0 (Not at all) to 4 (Very Great Extent). | S | T | T2 - T13 |
| Subjective norms | The modified Subjective Norms Measure used in previous BASIS studies and based on guidelines for developing reliable and valid measures of Theory of Planned Behavior (TPB) constructs, [108, 109] includes 8 items examining injunctive and descriptive EBP implementation norms. Subjective norm scales have demonstratched acceptable reliability (alpha >.74). An example item would be “Teachers like me find the time to improve the implementation of classroom management practices”. Respondents indicate the extent to which they agree with each statement on a scale of 0 (Strongly Disagree) to 5 (Strongly Agree). | S | T | T2 - T13 |
| Self-efficacy | The General Perceived Self-Efficacy Scale: A modified version of the Teacher Self-Efficacy Scale will be used to assess educators’ self-efficacy beliefs. This scale includes 10-items that assess teachers’ confidence and self-efficacy regarding teaching practices. The measure has demonstrated sufficient internal consistency, test-retest, and criterion validity [110]. This measure will also be used to assess maintenance self-efficacy after teachers initiate the implementation of PGD. Example items include, “If I am in trouble, I can think of a good solution”, and “ I can handle whatever comes my way”. Respondents indicate the extent to which they think each statement is true on a scale of 1 (Not at all) to 4 (Exactly true). | S | T | T2 |
| Maintenance self-efficacy | The Maintenance Self-Efficacy scale is a study-developed measure that is based on the General Perceived Self-Efficacy Scale [110] and has been used in other BASIS studies. It assesses to which extent educators feel confident that they can continue to implement PGD practices, given the many factors that could affect their ability to sustain implementation. Respondents will indicate how confident they would feel in their ability to implement the stated task on a scale of 0 (Not Confident at All) to 5 (Totally Confident) in the face of several difficulties specific to PGD. | S | T | T5 - T13 |
| Action self-efficacy | The Action Self-Efficacy Scale is a study-developed measure, also based on the General Perceived Self-Efficacy Scale [110] and has been used in other BASIS studies. It will be used to assess educators’ action self-efficacy within the school context. It takes into consideration factors such as relationships with colleagues, available resources, and demands on time. It includes items such as, “I feel confident that I can teach students how to behave responsibly in the classroom by:  ● Creating awareness of student expectations for classroom activities and transitions  ● Creating awareness of student expectations for classroom routines and policies”  Respondents would have to indicate how confident they would feel in their ability to implement the stated task on a scale of 0 (Not Confident at All) to 5 (Totally Confident). | S | T | T2 - T13 |
| Intentions to implement | The Modified-Intentions to Use Scale is based on work investigating practitioners’ intention to adhere to measurement-based care [111]. The scale includes 5-items modified to be consistent with educators’ intentions to use SEB EBPs. Our prior work demonstrates evidence of reliability/validity [47]. Respondents respond to the question on a scale of 0 (No intentions to implement at all) to 3 (Strong intentions to implement). | S | T | T4 - T13 |
| Outcome expectancy | Outcome expectancies are defined as beliefs about consequences (positive or negative) of engaging in a behavior. The outcome expectancy question will be used to assess educators’ beliefs that the implementation of classroom management strategies would lead to positive outcomes. An example time is, “If I implement classroom management practices frequently everyday, then my classroom will have positive outcomes”. Respondents indicate the extent to which they agree with each statement on a scale of 0 (Strongly Disagree) to 5 (Strongly Agree). | S | T | T2 - T13 |
| Coping planning | The Coping Planning self-assessment is a study developed measure that will be used to assess the extent to which educators have made a concrete plan to keep using PGD strategies in difficult situations. Example items include, “I made a concrete plan to Put everything into an “if [obstacle], then [solution]” plans”, and “Generate solutions to the obstacles”. Respondents indicate if None are in place, One is in Place, Two are in Place, or All are in Place. | S | T | T4 - T13 |
| Action planning | The Action Planning measure is a study developed measure that assesses the extent to which educators have a concrete plan to when, where and how to implement PGD practices. Respondents indicate if None are in place, One is in Place, Two are in Place, or All are in Place. | S | T | T4 - T13 |
| General implement-ation behaviors | The school-adapted version of the Implementation Citizenship Behavior Scale [112] consists of 10 items that capture commitment to EBP by keeping informed about the EBP being implemented and supporting colleagues to meet EBP standards. In the school context, both subscales have demonstrated strong inter-item consistency (α = .95 and .94). | S | T | T5 - T13 |
| *Organizational Factors* | | | | |
| Implement-ation leadership | The School Implementation Leadership Scale (SILS) [113] has 21 items loading onto 7 subscales: Proactive, Knowledgeable, Supportive, Perseverant, Communication, Vision, and Available. Subscale internal consistencies range from 0.91 to 0.96, and scores correlate with other leadership measures. | S | T | T4, T12 |
| Implement-ation climate | The School Implementation Climate Scale (SICS) [114] includes 21 items loading onto 7 subscales: Focus on EBP, Educational Support for EBP, Recognition for EBP, Rewards for EBP, Use of Data to Support EBP, Existing Supports for EBP, and EBP Integration, with good internal consistency estimates (range: 0.81–0.90). | S | T | T4, T12 |
| *Implementation and Sustainment Outcomes* | | | | |
| PGD adoption and reach | Adoption is operationalized as the initiation of PGD in a classroom at any point during study participation, based on teacher self-report.  Reach will be calculated as the proportion of students in each classroom (out of those eligible based on whether their classroom teacher participated in PGD training) who received PGD practices. | S | T | T4 - T13 |
| PGD fidelity | The PGD Observation Form was developed to gather observational data on the fidelity with which active ingredients of the intervention are delivered. School administrative staff who are masked to the condition will gather data using the PGD Fidelity Observation Form. There will be three month-long observation periods throughout the school year, near the beginning, middle, and end. School administrative staff will observe each of the participating classrooms once during each month of observation.  Self-Report Checklist of Fidelity of Implementation. Guided by the Treatment Integrity Planning Protocol and input from the PGD developers, a self-report checklist will also be used to capture adherence to delivering core PGD practices as planned. Teachers will complete the checklist monthly in both years. | S, O | T, C | T4 - T13 |
| Cost | Cost estimates from time surveys, budget worksheets, and project records. | S | T | T7 - T13 |
| *Student Outcomes* | | | | |
| Classroom behavior outcomes | A modified Direct Behavior Rating (DBR) will be completed by teachers based on their own observations of academic engagement (i.e., actively or passively participating in the classroom activity) and disruptive behavior (i.e., student actions that interrupt regular school or classroom activity), averaging across the classroom [115]. Teachers will complete the ratings after an English/Language Arts period to maintain consistency across students. | S | T | T5 - T13 |
| Education outcomes | Classroom average standardized test scores, attendance rates, and disciplinary incidents (office discipline referrals, suspensions, expulsions) | R | T | T5-T13 |

Type of measure: S = survey, R = records, O = Observation. Informant: T = teacher, R = record, C = coach
